# Some Unutilized Resources of Civilisation

**Published:** 1895-11-02

**Authors:** 


### The Hospital

An Institutional Journal of

The Medical Sciences and Hospital Administration, with

AN EXTRA NURSING SUPPLEMENT.

Vol. XIX.—No. 475.] [Nov. 2, 1895.

### Some Unutilised Resources of Civilisation.

The optimist, it is said, believes that "everything happens for the best in this best of all possible worlds." The medical physiologist, on the other hand, far from finding it possible to take so rosy a view of things, is often tempted to think that the British nation has been endowed with an abnormal quantity of "original stupidity," so slow is it to utilise or even to perceive the many important discoveries of modern physiology and medicine. As a concrete example of this may be mentioned the use made of the Medical Officer of Health and the pay so frequently doled out to him.

A properly educated Medical Officer of Health—and every Medical Officer of Health who now enters the ranks of health officers is properly educated—is a person who could in ten years make an extraordinary difference in the health and prosperity of his neighbourhood if he had but a free hand. If Great Britain were mapped out into districts of suitable size; if every district had a competent Medical Officer of Health; if, in addition, supervising officers, analogous to bishops, were appointed over counties or over groups of districts; and if all these Medical Officers of Health were so paid as to be entirely independent of private practice, the change which would be wrought in the health conditions of the country in a very few years would be little short of miraculous.

What would such a change mean? It would not mean merely drains made perfect, sewage utilised, rivers purified, poisonous manufactures rendered innoxious, smoke consumed, and the like; but it would mean healthy homes for the poor, air and light let in everywhere, ample playgrounds, abundant outdoor life, and England, even with its teeming modern millions, turned into an infinitely "merrier" and more prosperous England than pre-Reformation England ever dreamed of. "All of which," says the timid man, "would cost money"; and "all of which," says the man of practical science, "would repay itself tenfold in a very brief period of time."

Good work can never be done on a large scale, and for a long time, for inferior pay. If the country desires Medical Officers of Health who shall really be responsible men of business, men who will take up their duties with intelligence and determination, it must pay for them. No doubt medical men can be found, and that by the hundred, who will accept any salary a County Council or a municipality may offer. But such persons are the last in the world to be entrusted with the responsible charge of the public health. As well might we turn parish clerks and organists into rectors and vicars, because they would be willing to accept clerical offices at reduced incomes. As an instance of the failure of County Councils to comprehend and utilise to the full the medico-physiological resources of civilisation may be mentioned an extraordinary advertisement which was recently sent to one of the medical papers by the Sussex County Council. The Council required a Medical Officer of Health, not for a district, but for a division of the county; and they gravely offered, by way of "honorarium," one hundred pounds a year and travelling expenses. Such a salary or "honorarium" was quite absurd. What they should have offered if they wished for a competent, strong-minded, responsible man of business was not a hundred but a thousand a year. For a salary of a thousand a year and a permanent position they would have had the pick of the younger members of the medical profession, and they would, by the appointment of a man worthy of a thousand a year, have conferred advantages upon the county worth many thousands a year.

The real question is this: Does the nation, or does it not, wish to take advantage of all the sanitary and physiological resources which modern medicine has made itself master of? Or does it, because it is entirely ignorant of sanitary and physiological science, wish to hug itself in the chains of its own ignorance, and to let the country be made the victim of innumerable insanitary ills simply because it refuses to pay a reasonable market price for the sanitary science which is everywhere offered to it? A hundred a year will purchase the services of a medical man whose services are not worth even that amount; for no man of capacity and self-respect will do the work required for such beggar's wages. A thousand a year, we repeat, would purchase the services of a man whose work would be worth many thousands a year to the county which should employ him. Can a competent coroner be found to work for nothing? Yet surely nobody would consider the services of a coroner, who merely investigates the causes of abnormal deaths, to be anything like so really important to the country as the services of the man whose business it is to conserve and improve national vigour and health. The truth is we have not taken the trouble, as a people, to understand the relative importance of all these questions. But we must take the trouble, and we must consent to be taught. If we are to hold our own as a nation in the tremendous all-round struggle with other nations which lies immediately before us, we must have an individual and a national physique which shall be not only the equal but the superior of the individual and national physique of other peoples. Failing this we are lost. How are all these things to be made living realities among us? In no other way but by the intelligent use of those manifold resources which modern physiological and medical science have so abundantly placed at our command.

